# Insufficient Sleep and Health Care Utilization: A Scoping Review

**DOI:** 10.1093/geroni/igab046.3050

**Published:** 2021-12-17

**Authors:** Kolade Olatunde, Susan Patton

**Affiliations:** 1 University of Arkansas, University of Arkansas, Arkansas, United States; 2 University of Arkansas, Fayetteville, Arkansas, United States

## Abstract

Insufficient sleep is a common problem among older adults with 26% over the age of 65 reporting less than seven hours sleep in a 24 hour time period. Evidence indicates that untreated sleep disorders are associated with osteoarthritis, heart disease, hypertension, diabetes, obesity, falls, decreased cognitive performance, and decreased health related quality of life in older adults. A scoping review was undertaken to determine what is known about the association between insufficient sleep or insomnia and health care utilization. The Joanna Briggs Institute, Methodology for JBI Scoping Reviews was used to guide the review. Searches were conducted in PubMed, HINARI, Google Scholar and Cochrane databases. Twenty nine studies were included. Overall, the review indicates that reduced sleep is associated with a greater odds of difficulties in daily activities, higher rates of health care utilization and costs, and poly pharmacy. Findings also reveal sociodemographic and geographic variations in prevalence of healthy sleep duration. Although the majority of studies focused on the causes and consequences of insomnia and recommended clinical and behavioral health promotion interventions, there is a gap in studies related to the public health or economic impact of insufficient sleep. Research in this area will provide perspectives on the need to raise awareness of the importance of sleep and to incorporate the awareness into policies that improve sleep health.

